# Supplementary material for: Immunogenic Properties of Recombinant Enzymes from Bothrops ammodytoides towards the Generation of Neutralizing Antibodies against Its Own Venom
Source: Toxins (Basel). 2019 Dec 2;11(12):702. doi: 10.3390/toxins11120702 (PMC6949999; doi:10.3390/toxins11120702)
Supplement: Supplementary file 1 [file toxins-11-00702-s001.pdf]

Supplementary Materials: Immunogenic Properties of Recombinant Enzymes from Bothrops Ammodytoides Towards the Generation of Neutralizing Antibodies against Its Own Venom

Herlinda Clement, Ligia Luz Corrales-García, Damaris Bolaños, Gerardo Corzo and Elba Villegas

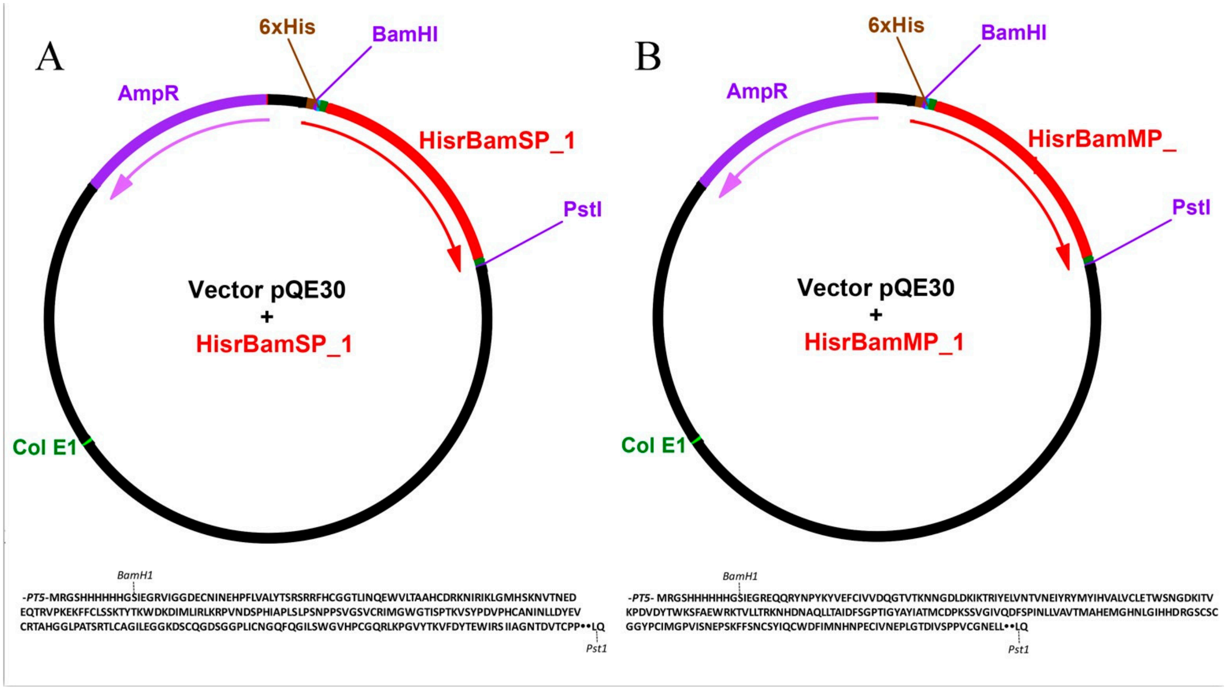

Figure S1. Genetic construction used for the expression of the recombinant rBamSP_1 and rBamMP_1, and the structural elements added to the sequence of the recombinant toxin are shown (the BamHI and PstI sites as well as the stop codons are underlined).

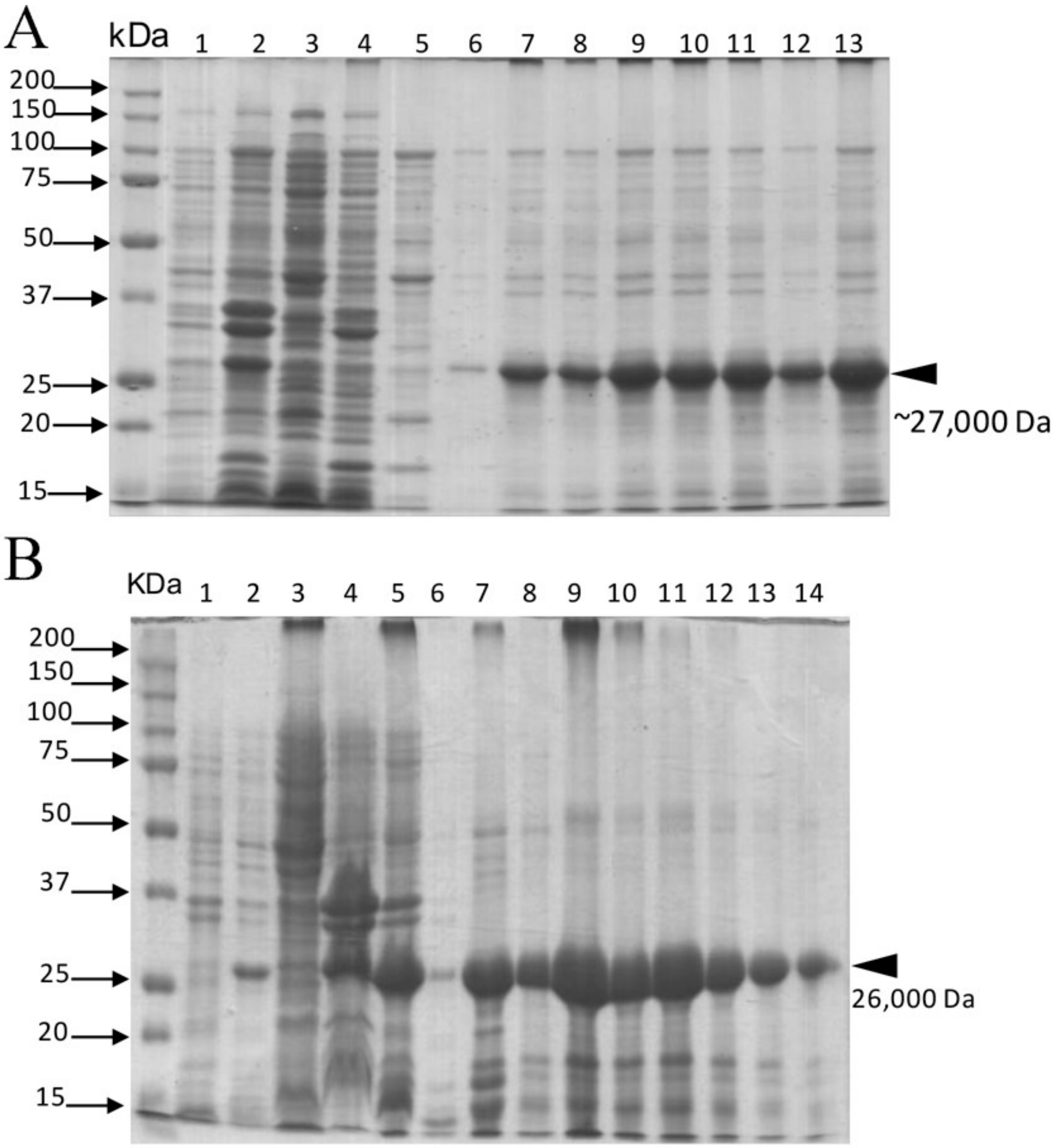

Figure S2. SDS-PAGE of rBamSP_1 and rBamMP_1 expressed in *E. coli* cells. A) kDa - Molecular weight markers; lane 1, cells without IPTG; lane 2, cells with IPTG; lane 3, supernatant; lane 4, unbound proteins; lane 5, first wash; lane 6, second wash; lanes 7-13, protein elutions. B) kDa - Molecular weight markers; lane 1, cells without IPTG; lane 2, cells with IPTG; lane 3, supernatant; lane 4, inclusion bodies; lane 5, unbound proteins; lane 6, first wash; lane 7, second wash; lanes 8-14, protein elutions.

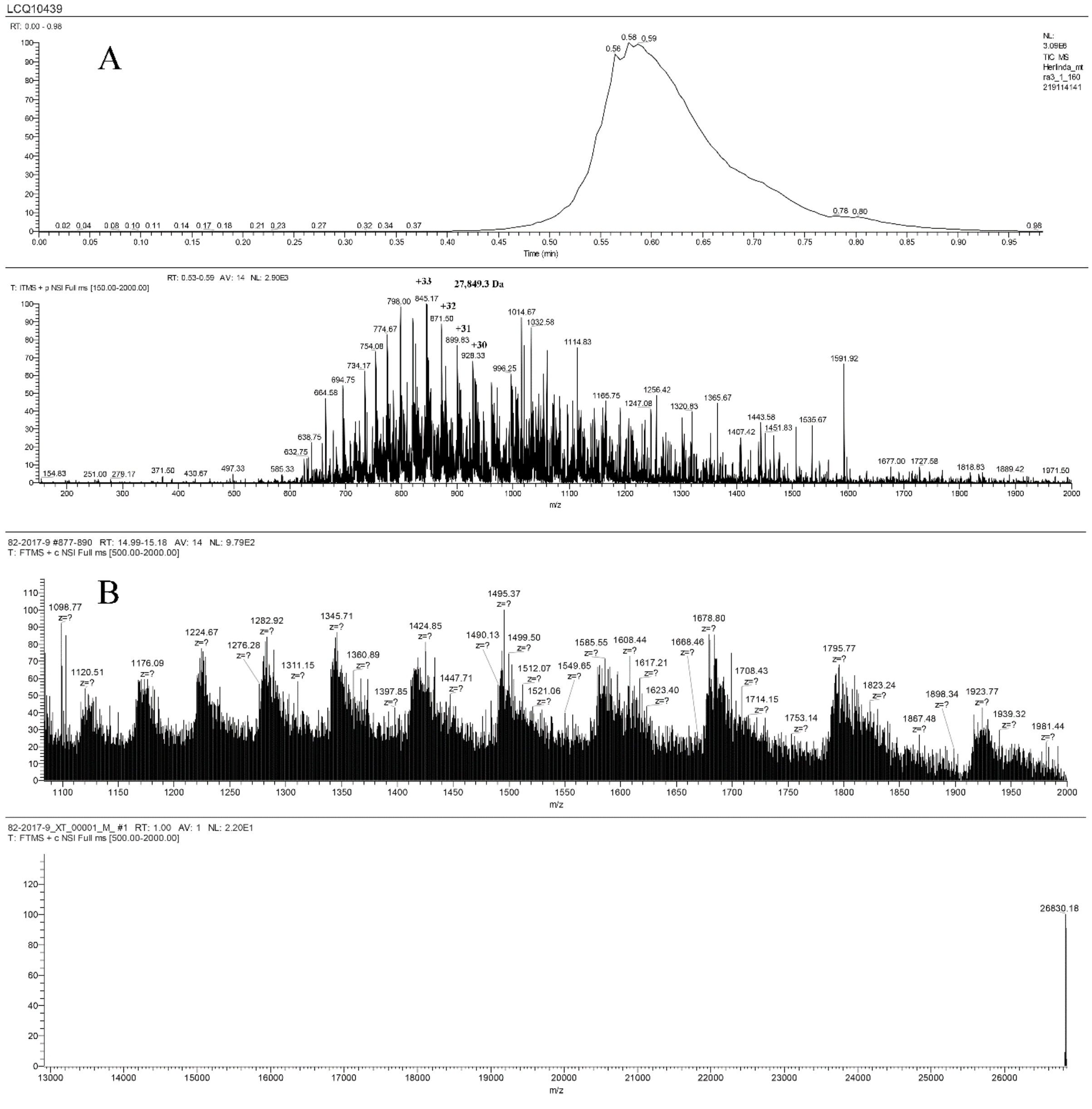

Figure S3. Mass spectrum of the recombinant proteins. A) rBamSP_1, the top spectrum comprises the raw data showing the total ion current (time vs. intensity), the bottom spectrum is the deconvoluted spectrum on the true mass scale after Xcalibur Windows NT PC data system processing. B) rBamMP_1, the top spectrum comprises the raw data showing the total ion current according to the charge ionization, the bottom spectrum is the deconvoluted spectrum also processed by Xcalibur Data Acquisition and Interpretation Software (see Materials and Methods).

Oligo Fw BamHI-FXa-SP_1
GGA TCC ATC GAG GGA AGG GTC ATT GGA GGT GAT GAA TGT (39nt) Tm= 60
G S I E G R V I G G D E C
Oligo Rv Stop-Stop-PstI-SP_1
CTG CAG TTA CTA CGG GGG GCA GGT CAC ATC (30nt) Tm=62
Q L * * P P C T V D

Oligo Fw BamHI-FXa-MP_1
GGA TCC ATC GAG GGA AGG GAA CAA CAA AGA TAT AAC CCC (39nt) Tm= 56
G S I E G R E Q Q R Y N P
Oligo Rv Metalo
CTG CAG TTA CTA CAA AAG TTC ATT TCC ACA AAC (30nt) Tm= 54
Q L * * L L E N G C V

Figure S4. The first forward and the last reverse oligonucleotides used for rBamSP_1 and rBamMP_1 assembly.

Table S1. Identification of proteins of by tandem mass spectrometry (MS/MS) indicating the percentage coverage.

Identified Proteins	Accession Number	kDa	Percentage of Coverage			
			Lane1	Lane2	Lane3	Lane4
Acidic phospholipase A2 BmooPLA2 OS=Bothrops moojeni OX=98334 PE=1 SV=1	PA2A_BOTMO	16			19	
Acidic phospholipase A2 OS=Bothrops ammodytoides	PA2A_BOTAM	14		42	20	
BATXCRISP1 OS=Bothrops atrox OX=8725 PE=2 SV=1	A0A1L8D673_BOTAT (+1)	27		21	11	
BATXSVSP7 OS=Bothrops atrox OX=8725 PE=2 SV=1	A0A1L8D5U8_BOTAT	28			23	
BATXSVSP10 OS=Bothrops atrox OX=8725 PE=2 SV=1	A0A1L8D5U9_BOTAT	29			32	
BATXSVSP18 OS=Bothrops atrox OX=8725 PE=2 SV=1	A0A1L8D5U3_BOTAT	28			11	
BATXSVSP20 OS=Bothrops atrox OX=8725 PE=2 SV=1	A0A1L8D5T9_BOTAT	28			26	
L-amino-acid oxidase (Fragment) OS=Bothrops pauloensis OX=1042543 PE=1 SV=1	OXLA_BOTPA	57				13

Snake venom serine protease HS114 OS=Bothrops jararaca OX=8724 PE=1 SV=1	VSP14_BOTJA	28			38	
Thrombin-like enzyme KN-BJ 2 OS=Bothrops jararaca OX=8724 PE=1 SV=1	VSP2_BOTJA	28		4.3	34	4.3
Venom nerve growth factor OS=Bothrops jararacussu OX=8726 GN=NGF PE=2 SV=1	NGFV_BOTJR	27	12			
Zinc metalloproteinase-disintegrin-like bothropasin OS=Bothrops jararaca OX=872.	VM3BP_BOTJA	68				5.8

[1]Samples were reduced with dithiothreitol (Sigma-Aldrich; St Louis, MO, USA), rented with iodoacetamide (Sigma-Aldrich) and digested "in solution" with Trypsin (Promega Sequencing Grade Modified Trypsin; Madison, WI, USA). A 50 mM solution of ammonium bicarbonate (pH 8.2) was used in the sample with trypsin and the incubation was 18 hw at a temperature of 37C. The peptides produced by enzymatic cleavage were desalted with Zip Tip C18 (Millipore; Billerica, MA, USA) and applied in an LC-MS (Liquid Chromatography-Mass Spectrometry) system composed of an EASY-nLC II nanoflow pump (Thermo-Fisher Co .; San Jose, CA, USA) coupled to a LTQ-Orbitrap Velos mass spectrometer (Thermo-Fisher Co., San Jose, CA, USA) with a nano-electrospray (ESI) ionization source. Total ion scanning (Full Scan) was performed on the Orbitrap analyzer with a resolution power of mass (RP Power; RP = m / FWHM) of 60,000. Peptide fragmentation was performed using the methods of CID (Collision-Induced Dissociation) and HCD (High-energy Collision Dissociation). All spectra were acquired in positive detection mode. The execution and capture of the fragmentation data were performed depending on the total ion scan according to the pre-determined charges (only ions with z2 +, z3 + and z4 + charges were fragmented) with an isolation width of 2.0 (m / z), normalized collision energy of 35 arbitrary units, Q activation of 0.250, activation time of 10 milliseconds and maximum injection time of 10 milliseconds per micro-scan. During the automatic capture of data the dynamic ion exclusion was used: (i) 500 ion exclusion list, (ii) 30 seconds pre-exclusion time and (iii) 70 seconds exclusion time. The PDB search and protein identification was performed with the spectrometric data in .raw format in the Proteome Discoverer 1.4.1.14 program (Thermo-Fisher Co., San Jose, CA, USA) through the Sequest HT search engine. For the identity search, the bothrops.fasta (UniProt) protein database was used.
